# Predictors of Diagnostic Yield in Shape-Sensing Robotic-Assisted Bronchoscopy (ssRAB): A Retrospective Single-Center Study

**DOI:** 10.3390/diagnostics16131954

**Published:** 2026-06-23

**Authors:** Hruy Menghesha, Jan Arensmeyer, Philipp Feodorovici, Mark Coburn, Dirk Skowasch, Tatjana Dell, Julian Luetkens, Joachim Schmidt, Donatas Zalepugas

**Affiliations:** 1 Department of Thoracic Surgery, University Hospital Bonn, University of Bonn, 53127 Bonn, Germany; 2 Department of Thoracic Surgery, Helios Clinic Bonn/Rhein-Sieg, 53123 Bonn, Germany; 3 Bonn Surgical Technology Center (BOSTER), University Hospital Bonn, Joseph-Schumpeter-Allee 1, 53227 Bonn, Germany; 4 Department of Anesthesiology and Intensive Care Medicine, University Hospital Bonn, University of Bonn, 53127 Bonn, Germany; 5 Department of Internal Medicine II—Pneumology/Cardiology, University Hospital Bonn, University of Bonn, 53127 Bonn, Germany; 6 Department of Diagnostic and Interventional Radiology, University Hospital Bonn, University of Bonn, 53127 Bonn, Germany

**Keywords:** lung cancer screening, robotic bronchoscopy, shape sensing technology

## Abstract

**Background/Objectives:** Robotic-assisted bronchoscopy has emerged as an advanced technique for the evaluation of peripheral pulmonary lesions, offering improved navigation and targeting accuracy. While several studies investigating other diagnostic modalities have identified factors associated with higher diagnostic yield, such determinants remain poorly defined for shape-sensing robotic-assisted bronchoscopy (ssRAB). This study therefore aimed to identify predictors of diagnostic yield in robotic bronchoscopy. **Methods:** This retrospective single-center study included all consecutive patients who underwent ssRAB (ION^TM^ system, Intuitive Surgical, Sunnyvale, CA, USA) between August 2024 and March 2026. Lung nodules undergoing marker placement only or procedures performed without cone-beam CT (CBCT) guidance were excluded. Collected variables included demographic characteristics, lesion size, lesion density (solid, part-solid, ground-glass), biopsy modality, and number of biopsy samples obtained. Diagnostic yield was defined as a definitive pathological diagnosis of the target lesion. Predictors of diagnostic success were assessed using univariable logistic regression. **Results:** In total, 111 pulmonary nodules were included in the analysis. The overall diagnostic yield was 88.3% (98/111). The mean patient age was 64.94 ± 7.9 years, with a predominance of female patients (58.4%). No significant associations were observed between diagnostic yield and lesion size (odds ratio [OR] 1.014 per mm; *p* = 0.764), lesion density (*p* = 0.892), or biopsy instrument (*p* = 0.835). However, an increased number of biopsy samples showed a positive association with diagnostic yield, showing a statistical trend (OR 1.22 per additional sample; *p* = 0.084). **Conclusions:** Robotic-assisted bronchoscopy provides a high diagnostic yield for peripheral pulmonary lesions. The number of biopsy samples appears to be the most relevant modifiable factor influencing diagnostic success, underscoring the importance of adequate tissue acquisition. In contrast, lesion characteristics and biopsy modality did not significantly affect outcomes in this cohort.

## 1. Introduction

The increasing detection of pulmonary nodules, driven by the widespread use of high-resolution computed tomography and lung cancer screening programs, poses a growing diagnostic challenge in clinical practice [1,2]. Accurate tissue diagnosis of these lesions is essential for appropriate patient management, particularly in the context of early lung cancer detection and the expanding role of personalized oncologic therapies [3,4,5]. However, obtaining representative tissue samples from peripheral pulmonary lesions remains technically demanding [5,6,7,8].

Conventional bronchoscopic techniques are often limited by restricted reach, insufficient stability, and suboptimal confirmation of tool position within the target lesion, especially in small or peripherally located nodules [7,9,10]. Diagnostic yields of conventional bronchoscopic approaches for peripheral pulmonary lesions have been reported to range between approximately 60% and 75%, depending on lesion characteristics and the navigational technology used. In contrast, transthoracic needle biopsy generally achieves higher diagnostic accuracy but is associated with pneumothorax rates of up to 20–25% [6,11].

Shape-sensing robotic-assisted bronchoscopy (ssRAB) has recently emerged as an advanced navigational platform designed to overcome these limitations [12,13]. By providing enhanced maneuverability, improved catheter stability, and more precise navigation to distal airways, this technology has the potential to increase diagnostic accuracy while maintaining a favorable safety profile [14,15]. Early clinical studies have reported promising diagnostic yields; however, data on factors influencing procedural success remain limited [16].

Although several studies have evaluated predictors of diagnostic yield in ssRAB, reported associations have been inconsistent. Factors such as lesion size, lesion characteristics, and procedural variables have shown varying effects across different cohorts [17,18]. In particular, potentially modifiable procedural factors, including the number of biopsy samples obtained, remain insufficiently characterized [19,20].

Therefore, the aim of the present study was to evaluate the diagnostic performance of ssRAB in a real-world clinical setting and to identify predictors of diagnostic yield, with a particular focus on lesion-related and procedure-related factors.

## 2. Materials and Methods

### 2.1. Study Design and Patient Selection

**Figure 1:**
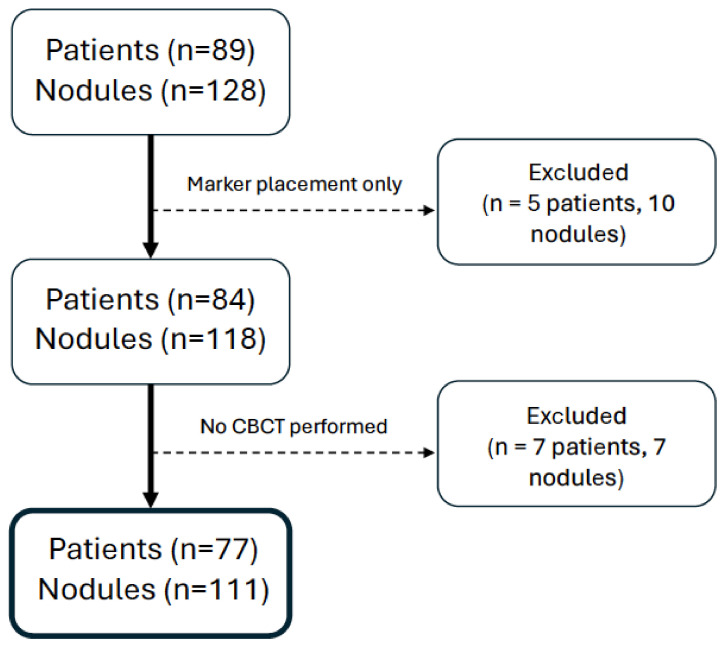
Flow diagram of patient and nodule selection.

This retrospective cohort study included all consecutive patients undergoing ssRAB (ION^TM^ system, Intuitive Surgical, Sunnyvale, CA, USA) at our tertiary thoracic surgical center between August 2024 and March 2026. All adult patients with detected lung nodules referred for diagnostic bronchoscopy were considered. Patients undergoing marker placement only or procedures performed without CBCT guidance were excluded (Figure 1).

The study was approved by the ethics board (2026-205-BO) on 21 April 2026. All patient data included in this analysis were fully available at the time of data collection. The data were obtained exclusively from the institution’s electronic medical records. Due to the retrospective nature of the study and the complete anonymization of all datasets, individual patient consent was not required in accordance with applicable data protection regulations and ethical guidelines. The study was conducted in accordance with the ethical principles outlined in the Declaration of Helsinki.

### 2.2. Preoperative Preparation

All cases were discussed by a multidisciplinary tumor board. Patients selected for tissue diagnosis underwent preoperative clinical and functional assessment according to institutional standards before ssRAB was performed.

### 2.3. Procedural Workflow

**Figure 2:**
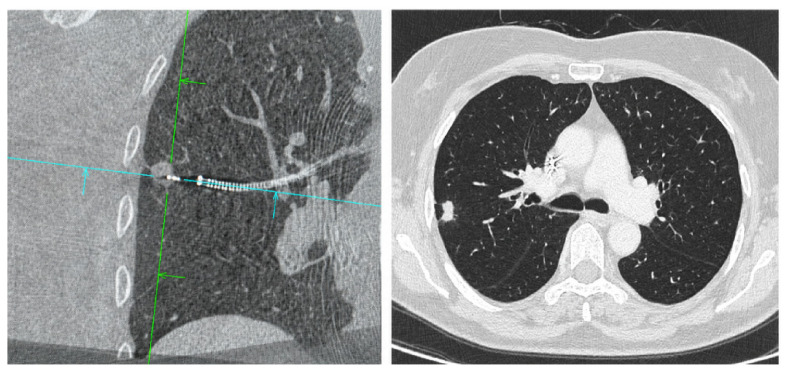
Representative intraprocedural and radiological imaging of a right upper lobe pulmonary nodule. (**Left**) Tool-in-lesion confirmation with the cryoprobe accurately positioned within the target lesion during shape-sensing robotic-assisted bronchoscopy (ssRAB) performed with the ION^TM^ Endoluminal System. (**Right**) Axial chest CT image in the lung window setting demonstrating the corresponding pulmonary nodule in the right upper lobe.

**Figure 3:**
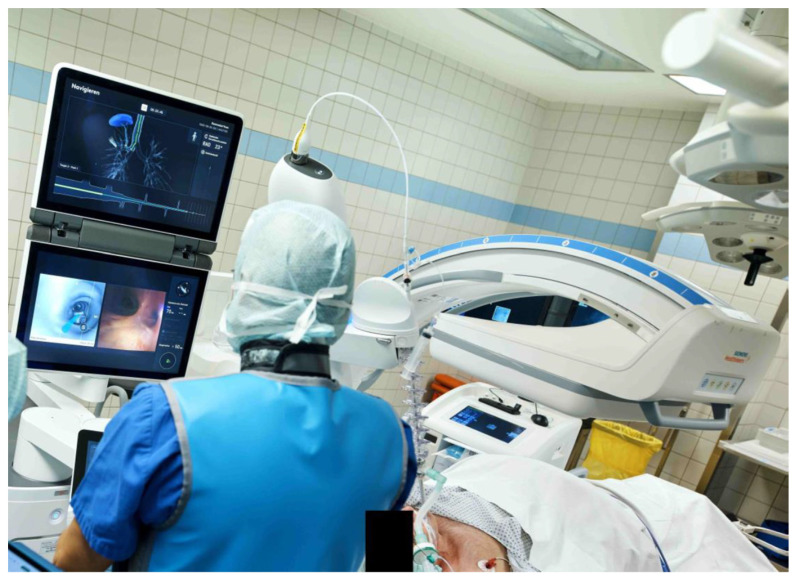
Operating room setup with ION ssRAB and CBCT.

All procedures were performed under general anesthesia with endotracheal intubation and continuous intraoperative management by an attending anesthesiologist. All ssRABs were conducted by an attending thoracic surgeon using the ION^TM^ system (Intuitive Surgical, Sunnyvale, CA, USA). Target navigation was based on preprocedural planning using the PlanPoint^TM^ software version OS1v5.1.1.2f65f5f04 (Intuitive Surgical, Sunnyvale, CA, USA), which generates CT-derived virtual airway maps to enable precise access to peripheral pulmonary lesions. In all cases included in the present analysis, lesion localization and tool-in-lesion confirmation (Figure 2) were performed using cone-beam computed tomography (CBCT), ensuring a standardized imaging-guided approach throughout the study cohort (Figure 3). Biopsy instruments were selected at the operator’s discretion according to lesion characteristics and included needle, forceps, cryoprobes, or a combination of forceps and cryoprobes. Intraoperative frozen section analysis was performed when clinically indicated. In cases of confirmed or highly suspected malignancy, subsequent surgical resection was carried out during the same anesthetic session.

Procedure-related adverse events were classified according to the Nashville Delphi Consensus Statement and CTCAE criteria. Minor bleeding was defined as bleeding requiring no intervention beyond routine suctioning and wedging maneuvers.

### 2.4. Histopathological Workup

Tissue samples obtained during robotic bronchoscopy using the ION^TM^ system were either subjected to intraoperative frozen section analysis or directly processed for definitive histopathological evaluation, depending on the planned clinical management. Frozen section analysis was performed in cases where immediate surgical decision-making was anticipated. In contrast, when a single-stage surgical approach was not intended, specimens were sent directly for standard processing with formalin fixation and paraffin embedding (FFPE). In patients who subsequently underwent definitive surgical resection following biopsy confirmation, resected specimens were processed using the same FFPE protocol. Final histopathological diagnoses were established in accordance with the most recent World Health Organization (WHO) classification criteria.

### 2.5. Outcome Measures and Statistical Analysis

Baseline characteristics were summarized using descriptive statistics. Continuous variables are presented as median with range or mean with standard deviation, while categorical variables are reported as absolute numbers and percentages. The primary outcome of this study was the diagnostic yield of ssRAB. The definition of diagnostic yield was adopted from the American Thoracic Society and American College of Chest Physicians consensus statement and was defined as the proportion of procedures resulting in a specific malignant or benign diagnosis of the target lesion [17]. Secondary analyses focused on identifying factors associated with diagnostic success. Potential predictors—including lesion size, lesion density (solid, part-solid, pure ground-glass), biopsy modality, and the number of biopsy samples obtained—were evaluated using univariable logistic regression analysis. Due to the limited number of non-diagnostic cases and the resulting risk of model overfitting, multivariable logistic regression analysis was not performed. Instead, potential predictors of diagnostic yield were assessed using univariable logistic regression to ensure model stability and avoid unreliable estimates. To improve statistical robustness and reduce model complexity, selected categorical variables were dichotomized. Lesion density was grouped into solid versus subsolid lesions (including part-solid and pure ground-glass), reflecting clinically relevant distinctions. Similarly, biopsy modality was dichotomized into cryobiopsy versus non-cryobiopsy techniques to avoid sparse data bias and unstable estimates observed in the multi-category analysis. Results are reported as odds ratios (ORs) with corresponding *p*-values. A *p*-value < 0.05 was considered statistically significant. Statistical analysis was conducted using IBM SPSS Statistics, Version 29.0 (IBM Corp., Armonk, NY, USA).

## 3. Results

### 3.1. Patient Characteristics

A total of 77 patients with 111 pulmonary nodules were included in the analysis. The mean age was 64.9 ± 7.9 years, and 45 patients (58.4%) were female. Regarding smoking status, 27 patients (35.1%) were current smokers, 27 (35.1%) were former smokers, and 23 (29.9%) had never smoked. The mean cumulative smoking exposure was 31.5 ± 17.0 pack-years. The mean body mass index was 27.6 ± 5.9 kg/m^2^ (Table 1).

### 3.2. Lesion Characteristics

Most lesions were located in the peripheral third of the lung (82.9%), followed by the middle (14.4%) and central third (2.7%). With respect to lobar distribution, lesions were most frequently found in the right upper lobe (36.9%), followed by the left lower lobe (26.1%), left upper lobe (24.3%), right lower lobe (9.0%), and middle lobe (3.6%). The mean lesion size was 10.8 ± 6.9 mm.

The majority of nodules were solid (78.4%), while 9.0% were part-solid and 12.6% presented as pure ground-glass opacities (Table 1).

### 3.3. Procedural Characteristics

All procedures were performed using CBCT guidance (111/111, 100%). Regarding biopsy modality, cryobiopsy was the most frequently used technique (65/111, 58.6%), followed by forceps biopsy (35/111, 31.5%), combined cryobiopsy and forceps (9/111, 8.1%), and needle biopsy (2/111, 1.8%). The mean number of biopsy samples per nodule was 7.1 ± 2.5. The mean procedure time was 38.6 ± 17.4 min. The overall diagnostic yield was 88.3% (98/111). No serious adverse events occurred. Minor bleeding was observed in one case (1/111, 0.9%) (Table 1).

### 3.4. Analysis of Factors Associated with Diagnostic Yield

Univariable logistic regression analysis was performed to evaluate potential predictors of diagnostic yield. Lesion size was not significantly associated with diagnostic success (OR 1.014 per mm, 95% CI 0.928–1.108, *p* = 0.764). Lesion density showed no significant impact on diagnostic yield, both in the initial three-category analysis (*p* = 0.941) and after dichotomization into solid and subsolid lesions (OR 0.91, 95% CI 0.23–3.60, *p* = 0.892). Biopsy modality was not significantly associated with diagnostic yield (*p* = 0.486). Due to sparse data and model instability in the multi-category analysis, subgroup-specific estimates were not interpreted. After dichotomization, the use of cryobiopsy was likewise not associated with diagnostic success (OR 1.14, 95% CI 0.33–3.99, *p* = 0.835). A trend toward higher diagnostic yield was observed with an increasing number of biopsy samples (OR 1.22 per additional sample, 95% CI 0.97–1.53, *p* = 0.084) (Table 2).

## 4. Discussion

In this retrospective single-center study, robotic-assisted bronchoscopy achieved a high overall diagnostic yield of 88.3% in the evaluation of peripheral pulmonary lesions. Among the variables assessed, neither lesion size, lesion density, nor biopsy modality showed a statistically significant association with diagnostic success. In contrast, the number of biopsy samples obtained emerged as the most relevant procedural variable, showing a clear trend toward higher diagnostic yield with increasing sample number. Taken together, these findings suggest that, within a highly standardized robotic bronchoscopy workflow, procedural sampling intensity may be more important than lesion-related characteristics in determining diagnostic success.

The overall diagnostic yield observed in the present cohort underscores the clinical value of robotic-assisted bronchoscopy for the workup of peripheral pulmonary nodules. Accurate diagnosis of such lesions remains challenging, particularly when lesions are small, located in the outer lung periphery, or exhibit subsolid morphology [21]. Conventional bronchoscopic approaches have historically been limited by restricted reach, suboptimal stability at the target, and imperfect confirmation of tool-in-lesion position [7,19]. Robotic bronchoscopy was developed to address several of these limitations by improving navigation, catheter stability, and distal maneuverability [12,22,23]. In our series, the high diagnostic yield supports the assumption that these technological advantages can translate into robust clinical performance in daily practice when procedures are integrated into a structured workflow and complemented by cone-beam CT confirmation.

One notable finding of this study is the lack of a significant association between lesion size and diagnostic yield. In many conventional diagnostic settings, lesion size is considered an important determinant of procedural success, with smaller lesions generally being more difficult to localize and sample adequately [24,25]. In our cohort, however, lesion size did not meaningfully influence the probability of obtaining a diagnostic result. This may indicate that the robotic platform, in combination with intraoperative imaging confirmation, reduces the technical disadvantage typically associated with smaller lesions. Although this interpretation should be made cautiously, it raises the possibility that robotic bronchoscopy may attenuate the impact of classical anatomical predictors that are known to limit the performance of non-robotic approaches [26,27].

A similar pattern was observed for lesion density. Neither the initial three-category analysis nor the dichotomized comparison between solid and subsolid lesions revealed a significant association with diagnostic yield. From a clinical perspective, this is an important observation, as part-solid and pure ground-glass lesions are often considered more challenging targets due to their less distinct margins and potentially lower cellular yield [28,29]. In the present study, however, lesion density did not appear to influence the likelihood of diagnostic success. This may again reflect the contribution of advanced navigation and imaging guidance, which may compensate for some of the difficulties traditionally encountered in less dense lesions. At the same time, the wide confidence intervals indicate that these estimates should be interpreted with caution, and larger studies are needed before firm conclusions can be drawn regarding lesion density as an independent determinant of robotic bronchoscopy performance.

Previous studies evaluating predictors of diagnostic yield in robotic bronchoscopy have reported heterogeneous findings regarding the influence of lesion characteristics and procedural factors [30,31]. This variability suggests that the relative importance of individual predictors may differ across patient populations, procedural workflows, and study designs.

Biopsy modality also did not demonstrate a statistically significant impact on diagnostic yield. The initial multi-category analysis of different sampling instruments proved unstable because of sparse data and the low number of non-diagnostic cases, precluding meaningful interpretation of subgroup-specific estimates. After simplification of the analysis, cryobiopsy versus non-cryobiopsy sampling likewise showed no association with diagnostic success. This finding suggests that the overall success of the procedure may depend less on the specific instrument used and more on accurate targeting and adequate tissue acquisition once the lesion has been reached. Nevertheless, this result should not be interpreted as evidence of equivalence between instruments. Different biopsy tools may vary in specimen size, tissue architecture preservation, crush artifact, molecular adequacy, or complication profile, none of which were specifically addressed in the present analysis. Accordingly, our data support the conclusion that no clear effect on immediate diagnostic yield could be demonstrated, but they do not exclude clinically relevant differences in other outcome domains.

The most clinically meaningful observation of this study is the trend toward improved diagnostic yield with a higher number of biopsy samples. Although this association did not reach conventional statistical significance, the effect estimate was notable, with each additional biopsy increasing the odds of diagnostic success by approximately 22%. This trend is biologically plausible and clinically intuitive. Even when lesion targeting is accurate, sampling error remains possible, particularly in heterogeneous lesions with necrotic, fibrotic, or inflammatory components. Obtaining a greater number of samples may reduce this sampling error and increase the likelihood of capturing representative tissue. In this context, the number of biopsy samples may be viewed as a modifiable procedural factor and therefore of particular practical interest. While lesion size or density cannot be changed by the operator, the extent of tissue acquisition can be adapted intraoperatively. Our findings therefore suggest that, once a lesion has been successfully reached, maximizing tissue acquisition within a safe procedural framework may be among the most important strategies for improving diagnostic performance.

This observation has immediate procedural implications. In daily practice, there may be a tendency to limit the number of biopsies after early pathological confirmation or after apparently satisfactory initial sampling. However, particularly in peripheral lesions, a limited number of samples may increase the risk of a non-diagnostic result due to sampling heterogeneity [20,32,33]. Our results support a more deliberate sampling strategy, with particular emphasis on obtaining an adequate number of specimens whenever procedural safety permits. This may be especially relevant in the context of precision oncology, where tissue requirements increasingly extend beyond simple histological confirmation and include molecular profiling and biomarker assessment [34,35]. Although molecular adequacy was not assessed in the present study, the principle that more comprehensive tissue acquisition may improve downstream diagnostic utility is highly relevant.

The absence of statistically significant associations for most variables should not be interpreted as proof that these factors are clinically irrelevant. Rather, these results need to be considered in light of the study design and event distribution. The very high overall diagnostic yield, while clinically favorable, inevitably resulted in a small number of non-diagnostic cases. This limited the statistical power of regression analyses and reduced the ability to detect small to moderate effects.

## 5. Limitations

Several limitations of this study should be acknowledged. First, the retrospective single-center design inherently introduces the possibility of selection bias and limits external generalizability. Second, the sample size, although sufficient to describe clinical performance, was limited for predictive modelling, particularly given the small number of non-diagnostic procedures. Third, the study focused on immediate pathological diagnostic yield and did not systematically assess longer-term endpoints such as diagnostic accuracy relative to final surgical pathology, false-negative rates, or longitudinal radiographic follow-up in benign or non-diagnostic lesions. Fourth, procedural success in robotic bronchoscopy is influenced by multiple technical and anatomical variables that were not included in the present analysis, such as bronchus sign, lesion eccentricity, catheter-to-nodule relationship, respiratory motion, and precise tool-in-lesion confirmation metrics. Finally, biopsy modality was analyzed in simplified form and without detailed assessment of specimen quality, sample volume, or molecular adequacy.

### 5.1. Strengths

Despite these limitations, the present study has several strengths. It reflects real-world clinical practice in a thoracic surgery center using a consistent robotic bronchoscopy platform and a standardized imaging-supported procedural workflow. The exclusion of procedures performed without cone-beam CT guidance increased methodological homogeneity and reduced an important source of technical variation. In addition, the study addresses a clinically relevant and still insufficiently characterized question, namely which factors determine diagnostic success once robotic bronchoscopy is implemented in routine care. The results suggest that, under these conditions, classical lesion-related variables may be less influential than expected, while procedural factors related to tissue acquisition may become increasingly important.

### 5.2. Future Directions

Future studies should focus on larger multicenter cohorts to validate these findings and improve statistical power for multivariable modelling. Particular attention should be given to procedure-related variables that may be modifiable in practice, including number of samples, sequence of instruments, use of cryobiopsy, and confirmation strategies for lesion targeting. It would also be valuable to assess additional endpoints beyond immediate diagnostic yield, such as complication rates, adequacy for molecular testing, concordance with final surgical pathology, and cost-effectiveness. Such analyses may help define optimized procedural algorithms for robotic bronchoscopy in different lesion types and clinical settings.

### 5.3. Conclusions

In conclusion, robotic-assisted bronchoscopy provided a high diagnostic yield for peripheral pulmonary lesions in this cohort. The present findings suggest a potential shift in the determinants of bronchoscopic success: whereas lesion-related variables have traditionally been regarded as major limiting factors, robotic bronchoscopy may reduce their relevance by improving access and targeting precision. Under these conditions, procedural execution—particularly the adequacy of tissue acquisition—may become the key determinant of diagnostic success.

## Figures and Tables

**Table 1:** Baseline characteristics.

	Total
	*n* = 77 Patients
	*n* = 111 Nodules
**Patient characteristics**	
Age (years)	64.94 ± 7.94
Female (%)	45 (58.44%)
Smoking status (%)	
current	27 (35.06%)
former	27 (35.06%)
never	23 (29.88%)
Pack years	31.54 ± 16.99
BMI (kg/m^2^)	27.64 ± 5.94
**Lesion characteristics**	
Topographical data	
Peripheral third	92 (82.9%)
Middle third	16 (14.4%)
Central third	3 (2.7%)
Lesion Lobe	
RUL	41 (36.9%)
ML	4 (3.6%)
RLL	10 (9.0%)
LUL	27 (24.3%)
LLL	29 (26.1%)
Lesion size (mm)	10.77 ± 6.94
Lesion Density	
Solid	87 (78.4%)
Part-Solid	10 (9.0%)
GGO	14 (12.6%)
**Procedural characteristics**	
Imaging tool	
CBCT	111 (100%)
Biopsy tool (nodules)	
Needle	2 (1.8%)
Forceps	35 (31.5%)
Cryoprobe	65 (58.6%)
Cryoprobe + Forceps	9 (8.1%)
Number of biopsies per nodule	7.12 ± 2.48
Procedure time (min.)	38.6 ± 17.4
Diagnostic yield (%)	88.3
(Serious) Adverse Event	0%
Minor bleeding	1 (0.9%)

**Table 2:** Univariable logistic regression.

	Odds Ratio	95% C.I.	*p*-Value
Lesion size	1.014	0.928–1.108	0.764
Lesion density	0.909	0.229–3.604	0.892
Biopsy instrument	1.142	0.327–3.987	0.835
Number of biopsies per nodule	1.222	0.973–1.533	0.084

## Data Availability

The datasets generated during and/or analyzed during the current study are not publicly available due to security and ongoing research. The data underlying this article will be shared on reasonable request to the corresponding author.
